# Mutant K-Ras in Pancreatic Cancer: An Insight on the Role of Wild-Type N-Ras and K-Ras-Dependent Cell Cycle Regulation

**DOI:** 10.3390/cimb45030164

**Published:** 2023-03-17

**Authors:** Robert Ferguson, Karen Aughton, Anthony Evans, Victoria Shaw, Jane Armstrong, Adam Ware, Laura Bennett, Eithne Costello, William Greenhalf

**Affiliations:** Liverpool Experimental Cancer Medicine Centre, University of Liverpool, Liverpool L3 5TR, UK

**Keywords:** pancreatic ductal adenocarcinoma, K-Ras, N-Ras, G2 cyclins

## Abstract

The development of K-Ras independence may explain the failure of targeted therapy for pancreatic cancer (PC). In this paper, active N as well as K-Ras was shown in all human cell lines tested. In a cell line dependent on mutant K-Ras, it was shown that depleting K-Ras reduced total Ras activity, while cell lines described as independent had no significant decline in total Ras activity. The knockdown of N-Ras showed it had an important role in controlling the relative level of oxidative metabolism, but only K-Ras depletion caused a decrease in G2 cyclins. Proteasome inhibition reversed this, and other targets of APC/c were also decreased by K-Ras depletion. K-Ras depletion did not cause an increase in ubiquitinated G2 cyclins but instead caused exit from the G2 phase to slow relative to completion of the S-phase, suggesting that the mutant K-Ras may inhibit APC/c prior to anaphase and stabilise G2 cyclins independently of this. We propose that, during tumorigenesis, cancer cells expressing wild-type N-Ras protein are selected because the protein protects cancer cells from the deleterious effects of the cell cycle-independent induction of cyclins by mutant K-Ras. Mutation independence results when N-Ras activity becomes adequate to drive cell division, even in cells where K-Ras is inhibited.

## 1. Introduction

Despite a growing understanding of the molecular changes that result in pancreatic cancer, the disease remains largely intractable to chemotherapy; moreover, prognosis is among the worst for any malignancy, with a 5-year survival of approximately 10% [1]. Novel therapeutic approaches based on Small Molecule Kinase Inhibitors [2,3,4] and Immune Checkpoint Inhibitors (ICIs) [5] do offer some hope for some patients; however, the responsive populations have, to date, proved to be a very small minority of patients who are difficult to identify in advance of treatment (e.g., those patients who develop rash with erlotinib treatment [6] or the rare pancreatic cancer patients with microsatellite instability who respond to ICIs [7]). As well as the relatively low mutation burden in pancreatic cancer, that in itself makes pancreatic cancer less amenable to ICIs than other cancers, the most common mutations found in pancreatic cancer are mutations in the *KRAS* gene, which in itself downregulates MHC-I, further reducing the efficacy of this form of therapy [8].

The *KRAS* gene is mutated in most cases of Pancreatic Ductal Adenocarcinoma (PDAC), with estimates of over 90% [9]. Furthermore, mutant K-Ras is found in preneoplastic lesions such as Pancreatic Intraepithelial Neoplasias (PanINs) [10] and Intraductal Papillary Mucinous Neoplasias (IPMN) [11]. This has led to the assumption that the mutation of *KRAS* is a common initiating event in pancreatic tumorigenesis, which is supported by research on murine models [12]. The activation of mutant *KRAS* under its own promoter in pancreatic progenitor cells using Cre recombinase under the pdx promoter results in PanIN-like lesions and eventually cancer [12]. These progenitor (bud) cells give rise to islets as well as both acinar and ductal epithelial cells. The expression of mutant K-Ras directly in ductal [13] or acinar [14] cells is not usually sufficient to induce PanIN or PDAC development in mice. The activation of mutant K-Ras under its own promoter, using Cre recombinase under an acinar specific (elastase) promoter can reproduce pancreatic tumorigenesis, but only if K-Ras is expressed during development or following cerulean-induced pancreatitis [15]. This may require the induction of Acinar Ductal Metaplasia (ADM) via the increased expression of the Hippo signalling protein YAP1 [16]. Suggesting that the best established role of the K-Ras mutation, i.e., the induction of Cyclin D1 in G1 cells, is inadequate in itself to promote carcinogenesis. The rise and fall of cyclin levels drives the cell cycle. In metazoans, there is a sequential rise in G1 cyclins (e.g., Cyclin D1) in G1, followed by a rise in G2 cyclins (e.g., cyclin B1) in G2. Although, recent evidence has suggested that cyclins are redundant in respect of cell cycle progression (rise and fall of any single cyclin type or any combination of cyclins would achieve progression) [17]. In quiescent cells, Ras activity causes the production of G1 cyclins to start this process. However, once cells are cycling, the level of cyclin D1 is determined by its protein stability and not by transcriptional regulation via Ras [18].

Targeting K-Ras has a long tradition in pancreatic cancer research; however, agents have struggled to progress from the laboratory into clinical practice, and recent progress with KRAS-G12C inhibitors offers potential; moreover, these have been licensed in the treatment of lung cancer but not yet pancreatic cancer [19]. The lack of an effective small molecule inhibitor of mutant K-Ras, in general, might in part be explained by the ability of cancer cells to survive losing K-Ras once tumours are established [20]. K-Ras loss in cell lines and murine models can be compensated for by using other mutations (including over-expression of the *YAP1* gene) [21]. Some cell lines which retain oncogenic K-Ras have become independent of the mutation, although others remain addicted to the oncogene [22]. The independence of K-Ras has often been associated with YAP1 overexpression and a consequent expression profile that is consistent with a quasi-mesenchymal phenotype [21].

The progression from preneoplastic lesions to carcinoma following K-Ras mutation requires Epidermal Growth Factor Receptor (EGFR) activity [23], and oncogenic K-Ras increases the level of this receptor [24]. Mutation of p53 relieves the requirement for EGFR activation [23,25]; furthermore, inflammation in the absence of *TP53* leads to pancreatic cancer in mice without the requirement for K-Ras mutation [26].

This contrasts with other cancer types, where K-Ras mutation seems to negate the need for the stimulation of EGFR [27,28]. Co-dependence on EGFR and mutant K-Ras may reflect the fact that they can activate the cell cycle via cyclin D expression independently [29], and this is supported further by the observation that the inhibition of various forms of cyclin-dependent kinase (including CDK1 and 2) is equivalent in action to the inhibition of mutant K-Ras in cancers that are addicted to K-Ras [30].

K-Ras dependency in cell lines is associated with an expression pattern that is also closely associated with EGFR inhibition [22]. Other forms of Ras (N and H-Ras) are rarely mutated in PDAC [31]; nevertheless, both are expressed [32] and may increase EGFR levels and responsiveness to EGF [33]. Therefore, the knockdown of N and H-Ras may promote K-Ras addiction, which may explain why the high expression of N and H-Ras are associated with poorer prognosis [34]. However, it has been suggested that K-Ras activates both N and H-Ras via the AKT target endothelial nitric oxide synthase (eNOS) [35], in which case they would lie downstream of K-Ras and not be independent of it. Singh et al. provided evidence that the K-Ras addiction expression profile is negatively associated with epithelial–mesenchymal transformation (EMT) [22], such that addicted cells appear more epithelial. Only one of the 9 K-Ras-dependent cell lines tested by this group was reported to have an expression profile predictive of K-Ras independence [22]; this was the pancreatic line Suit-2, which is well known to be more mesenchymal than other pancreatic cell lines [36]. We set out to characterise how mutant K-Ras interacts with wild-type Ras isoforms to control proliferation in this cell line, as this may inform strategies for treatment that not only target different (more commonly occurring) mutations of K-Ras [37], but also target specific downstream and upstream partners of pan-Ras, such as SOS1 [38].

## 2. Materials and Methods

### 2.1. Cell Culture

Suit-2, PANC-1, MIA-Pa-Ca-2, BxPC3, and HeLa cell lines were cultured in RPMI 1640 with 10% FBS and 1% L-glutamine (100 mM/L). These cell lines were genotyped using the PowerPlex 16HS system (Promega, Chilworth, Southampton, UK). Primary mouse pancreatic cancer cell lines were obtained from Professor Michael Schmid (Liverpool University), who isolated them from tumours arising in Kras^LSL-G12D/+^, p53^R172H/+^, and Pdx1-Cre mice (KPC) using a protocol described by Olive et al., 2009 [39]. After 3–4 passages, primary KPC-derived murine pancreatic cancer cells were grown in DMEM medium supplemented with 10% fetal bovine serum, 1% Amphotericin B, penicillin (100 U/mL), streptomycin (100 µg/mL), and L-glutamine (10 mmol/L) (all from Sigma Aldrich, Gillingham, UK). Low passage (<10) KPC cells were used for these experiments. Confluent T75 flask of cells was used to generate cell lysates. Cells were washed and recovered in PBS and then scraped off the bottom of the flask using a cell scraper The cell suspension was spun at 1000× *g* for 10 min in a benchtop centrifuge. The cell pellet was stored at −80 °C until cell lysates were made for Western blotting.

### 2.2. siRNA Treatment

Cells were seeded at 0.5 × 10^5^ cells per well of a 6-well plate. The cells were transfected using Lipofectamine-2000 following the manufacturer’s protocol. siRNA was purchased from GE Healthcare/Dharmacon (Lafayette, CO, USA), and 10 nmoL of human *KRAS* (J-005069-10-0050), *HRAS* (L-004142-00-0010), or *CCND1* (L-003210-00-0010) siRNA was used, and/or 100 nmoL human *NRAS* (L-003919-00-0010) siRNA. The cells were harvested 48 h after siRNA treatment and stored as a cell pellet until required at −80 °C.

### 2.3. Western Blot Analysis

Lysates were prepared from pellets using RIPA buffer and sonication, following standard protocols. Western blots were run on precast Any KD gels (Bio-Rad Labororatories Co., Ltd., Hercules, CA, USA) in accordance with standard protocols and transferred using the Bio-Rad Turbo blotter high molecular weight program. K-Ras (F234, sc-30, 1:500), N-Ras (F155, sc-31, 1:1000), Cyclin A (H-432, sc-751, 1:1000), B (H-433, sc-752, 1:1000), and E (C-19, sc198, 1:1000) were all purchased from Santa Cruz Biotechnology (Heidelberg, Germany) and Cyclin D (ab137875, 1:1000) was purchased from AbCam (Cambridge, UK). RNA-induced silencing complex (RISC) free control was from GE healthcare/Dharmacon Lafayette, CO, USA (D-001600-01-05). The membranes were blocked in 5% milk Phosphate Buffered Saline with 0.1% Tween (PBST 0.1%) for three hours before being incubated with primary antibody in 5% milk, PBST 0.1% at 4 °C overnight. Six ten-minute washes in PBST 0.1% were followed by incubation of the membrane with an HRP conjugated secondary anti-mouse antibody (sc-2357). Following incubation with a secondary antibody, six ten-minute washes with PBST 0.1% were carried out, and the membrane was visualised using chemo-luminescence and developed (Western lightning plus ECL Life Technologies, Paisley #31985-062).

### 2.4. Active Ras Pulldown

After 48hr of siRNA treatment, cells were lysed and the active Ras was pulled down, using Raf as a substrate, following the instructions provided in the Active Ras pulldown kit (Life Technologies, Paisley, UK).

### 2.5. Ubiquitin Pulldown Assay

After 24 h of siRNA treatment, the cells were treated with Bortezomib (Santa Cruz Biotechnology, Heidelberg, Germany, sc-217785, 10 nM) or DMSO diluted in 500 µL of RPMI media. The cells were harvested 48 h after siRNA treatment and ubiquitinated proteins were pulled down using a polyubiquitin affinity column (Life Technologies, Paisley, UK) following the manufacturer’s protocol.

### 2.6. Seahorse Analysis

Cells were seeded in a seahorse 24-well plate (Seahorse Bioscience, Copenhagen, DK) at 6000 cells/well. The cells were then treated with siRNA as above; however, only 15 µL of the siRNA mix was added to each well. The cells were then left for 48 h at 37 °C, 5% CO_2_. The cells were run on the Seahorse Bioanalyzer (Seahorse Biosciences, North Billerica, MA, USA) following the manufacturer’s protocols for an aerobic stress test.

Seahorse data were reported as OCR/ECAR which provides a measure of relative oxidative against non-oxidative phosphorylation. OCR/ECAR was obtained for each test prior to the addition of any drug and then the value was divided by OCR/ECAR for a control that was run in parallel (to normalise to the plate). At least 4 usable technical repeats were carried out for each experiment and each experiment was performed in triplicate. Data were considered usable only if the stress test showed the predicted response to the drugs (i.e., if any of the agents gave no response or the opposite response to that expected with Antimycin A + Rotenone, Oligomycin, or FCCP, then the data for that run were considered unusable; this was binary in that it either worked or it did not. Technical problems included things such as a reagent not being added or being expired and not due to variability in the assay).

### 2.7. FACS Analysis

Cells were treated with siRNA as above and left for 48 h, Then, they were then harvested and spun at 500× *g* in a benchtop centrifuge before being washed with PBS and re-spun. The cell pellet was then resuspended in 70% ethanol and left at 4 °C for at least 16 h. The samples were then spun at 500× *g* for 5 min and the supernatant was discarded. The sample was resuspended in 500 µL Propidium Iodide (PI) (50 µg/mL) and 50 µL of RNase (10 mg/mL diluted 1:10 in PBS). The sample was then gently mixed before being transferred to an FACS tube. A minimum of 50,000 cells per treatment were analysed on a BD LSR Fortessa flow cytometer (Beckton Dickinson, Oxford, UK). Data were analysed with appropriate gating to exclude debris, doublets, and clumps using the software FlowJo version 7 (TreeStar, Inc., Ashland, OR, USA). Cell cycle analysis was performed on the FlowJo cell cycle analysis platform using the Dean Jett Fox model.

### 2.8. Edu Analysis

Cells were labelled with 10 nmoL Edu (Life Technologies, Paisley, UK) at either 24, 26, 28, 30, or 32 h post siRNA treatment. The cells were then grown until 48 h post siRNA treatment, when they were harvested and stained using Alexa Fluor 647 (Click-iT^®^ EdU Alexa Fluor^®^ 647 Flow Cytometry Assay Kit, Life Technologies, Paisley, UK C10424) and PI following the manufacturer’s protocols.

### 2.9. Transcriptional Analysis

The cells were treated with siRNA and harvested as described above. The cell pellet was lysed, and mRNA was extracted using an RNAeasy Protect Mini Kit (Qiagen Co., Ltd., Manchester, UK) following the manufacturer’s protocol. cDNA was made using a QuantiTech Reverse Transcription Kit (Qiagen Co., Ltd., Manchester, UK) following the protocol supplied by the manufacturer. The qPCR was then performed using K-Ras, Cyclin D, and GAPDH primers (Qiagen Ltd, Manchester, UK), using the Roche cyber green system on the Roche light cycler following the manufacturer’s instructions.

### 2.10. Statistics

All statistical analysis was performed using Statistical Package for the Social Sciences software (version 21.0 for Windows; SPSS, Inc., Chicago, IL, USA). Repeat measure data were tested using an independent sample T-test (significance quoted is 2-tailed). All data were obtained with at least three parallel analyses.

## 3. Results

**Figure 1:**
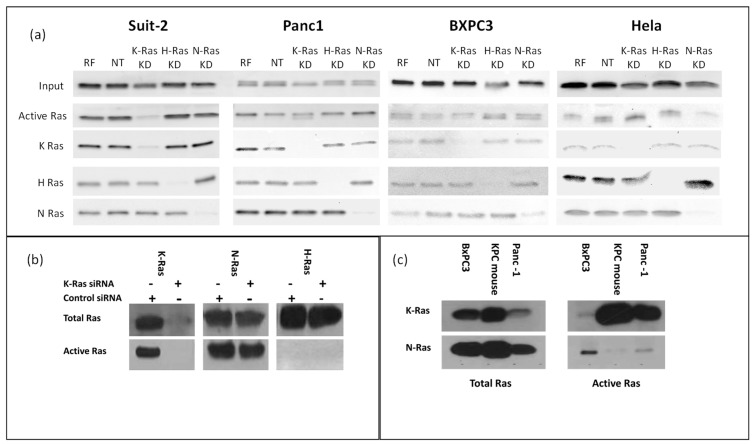
Total and active Ras isoforms in cancer cell lines: (**a**) Western blot analysis of total Ras in K-Ras mutation addicted (Suit-2) and independent cell lines (Panc1, BXPC3, HeLa). For each cell line, the upper panels show all Ras protein isotypes, and the lower panel shows active Ras isolated by pulldown with Raf, followed by panels of the total levels of each individual isotype. Cells were treated as shown with K-Ras, siRNA, or non-targeting control as indicated (RF = RISC Free, NT = Non-Targeting, KD = Knock Down). K-Ras depletion only depletes active Ras in the mutant Ras-dependent cell line Suit-2. (**b**) Western blot analysis of Suit-2 showing the presence of N-Ras in the upper panels and active N-Ras in the lower panel. Cells were treated as shown with K-Ras, siRNA, or non-targeting control. (**c**) Total Ras isoforms (left) or active Ras isolated by Raf pulldown (right). Cell line name is shown above the blot.

Western blotting confirmed that N-Ras, H-Ras, and K-Ras were expressed in all the pancreatic cancer cell lines tested (Figure 1a). All the cell lines had active Ras when tested for activity using binding to Raf and a pan-Ras antibody (Figure 1a). The BxPC3 cell line, which is a wild-type K-Ras pancreatic cell line, appears to have the lowest levels. To test whether this activity was due to K-Ras alone, each isoform was specifically knocked down with siRNA; moreover, the K-Ras-dependent cell line Suit-2 showed the most marked decrease in active Ras when K-Ras was knocked down. In the other cell lines, any decline in active Ras following K-Ras depletion was marginal (justifying our subsequent concentration on this specific cell line). The non-pancreatic cell line HeLa showed the greatest decline in Ras activity when N-Ras was specifically targeted (Figure 1a). When the specific activity of each Ras isotype was tested in the K-Ras-dependent cell line (Suit-2) using specific K, H, and N-Ras antibodies (Figure 1b), there was evidence of N as well as K-Ras activity. H-Ras activity was not seen, although this could reflect the sensitivity of the assay. The knockdown of K-Ras did not cause any noticeable change in N-Ras or the appearance of detectable H-Ras activity, suggesting that the residual Ras activity seen in Suit-2 after K-Ras knockdown was largely the basal level of N-Ras activity. The K-Ras wild-type cell line BxPC3 had substantial N-Ras activity, while N-Ras activity was also detectable (albeit at a relatively low level) in the K-Ras independent cell line Panc-1, which has the same p.G12D mutation as Suit-2 (Figure 1c). As neither of these cell lines is dependent on a K-Ras mutation, and since neither exhibits a loss of total Ras activity following K-Ras depletion (Figure 1a) and both have at least some N-Ras activity, it is reasonable to assume that isotype agnostic Ras activity can support cell division in these cell lines. In contrast, the primary mouse pancreatic cancer cell line isolated from tumours arising in the Kras^LSL-G12D/+^, p53^R172H/+^, Pdx1-Cre mice (KPC) mouse model [12] had little or no detectable N-Ras Raf-binding activity (Figure 1c). The KPC mice express mutant Ras in the progenitor cells of the pancreas, which differentiate into the cells of the mature pancreas, while the human cell lines have spontaneous K-Ras mutations occurring (presumably) in differentiated cells. In both forms of tumorigenesis, the mutation must be tolerated in pre-cancerous lesions. In the case of spontaneous mutations, toleration can result from selection (most cells will die, with the select few being adapted or mutated to accept the change), while, in the progenitor cells, toleration requires an innate characteristic, as most cells must survive for the pancreas to form. On this basis, the innate toleration might not require the expression of other (wild type) Ras isoforms, in contrast to a selected toleration.

### 3.1. Effect on Cyclins and The Cell Cycle of Knocking down Ras Species

**Figure 2:**
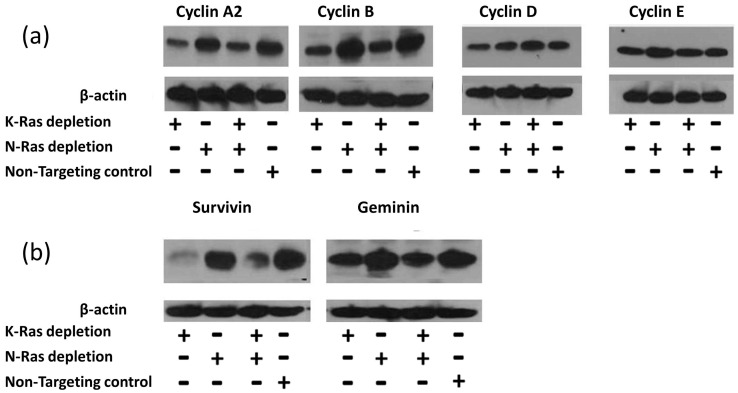
Effect of K-Ras depletion on G2 cyclins and other APC/c targets. (**a**) Western blot analysis of Suit-2 cells treated with K-Ras, N-Ras, or non-targeting siRNA showing the levels of cyclins. (**b**) Other targets of the APC/c complex and their levels following siRNA treatment.

siRNA-mediated depletion of K-Ras in Suit-2 cells resulted in a reduction in the main G2 cyclins (A2 and B1) with comparatively little effect on G1 cyclins (D1 and E) (Figure 2a). Depletion of N-Ras, either alone or in combination with depletion of K-Ras, had little effect on the levels of any of the cyclins (Figure 2a).

The regulation of G2 cyclins is usually considered to be carried out via the Anaphase Promoting Complex (APC/c) [40], although previous reports of EMT control via the G2 cyclin, A2, implied an alternative regulatory mechanism [41]. To determine if the inhibition of the APC/c complex could be responsible for the K-Ras-mediated stabilisation of G2 cyclins, other targets of APC/c were analysed; the levels of both survivin and geminin exhibited an equivalent relationship with K and N-Ras depletion (Figure 2b). The same relationship between survivin and K-Ras depletion has been reported previously [42].

**Figure 3:**
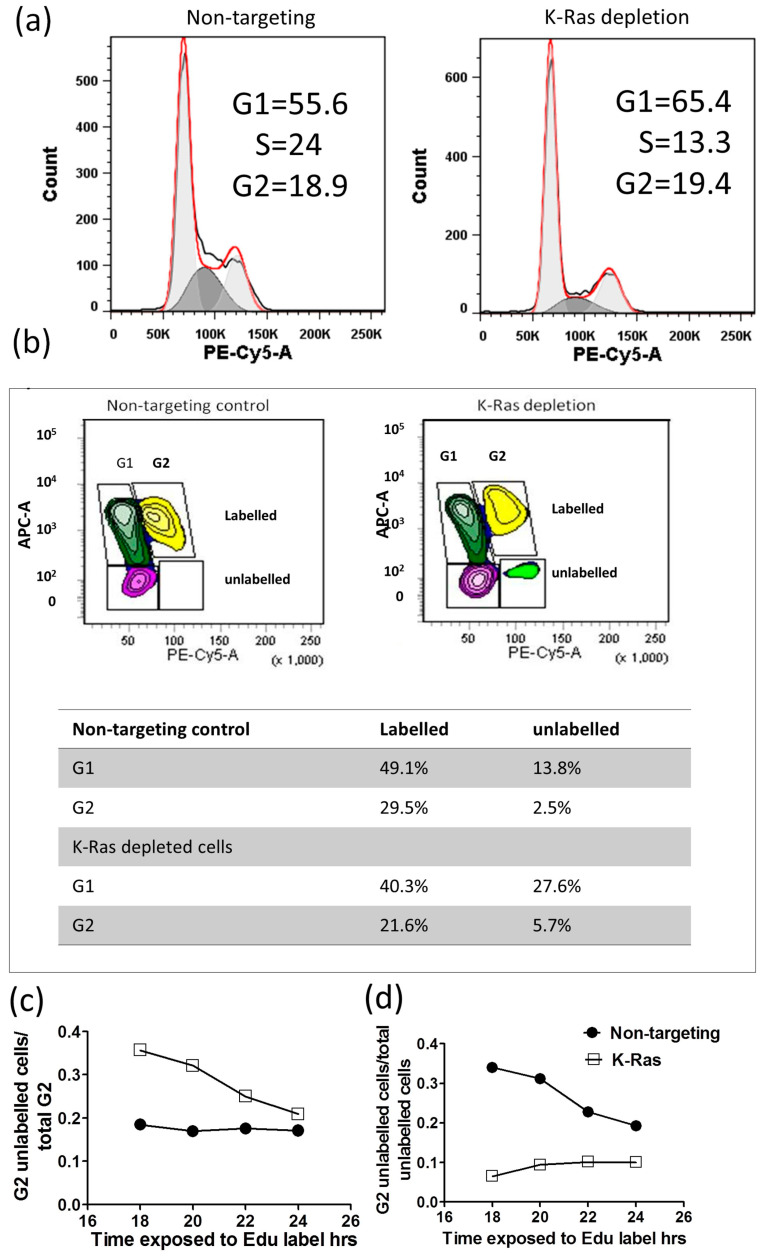
Cell cycle analysis following K-Ras depletion: (**a**) FACS analysis of the cell cycle using PI staining showing reduced S-phase but little change in G2 level. (**b**) An example of Edu analysis of the cell cycle showing that K-Ras depletion increases the percentage of unlabelled cells in G2. (**c**,**d**) Graphs showing how unlabelled G2 cells change with length of Edu labelling. (**c**) After 18 h of labelling, nearly 40% of K-Ras-depleted G2 cells are still unlabelled; however, this declines with labelling time as labelled cells enter from S-phase and unlabelled cells move towards G1. Without K-Ras depletion, the proportion of unlabelled cells seem to be stable (therefore, any entry of cells from S-phase is compensated for by disproportionate exit of the G2 of labelled cells compared to unlabelled). (**d**) With K-Ras depletion, the percentage of total unlabelled cells that are in G2 remains almost constant, suggesting that unlabelled cells are leaving G2 and entering G1 at a slower rate than unlabelled G1 cells are entering S-phase. Without K-Ras depletion, the unlabelled cells from G2 accumulate in G1 over time, suggesting that cells are exiting G2 at an equivalent or greater rate than cells are leaving G1.

The activation of the APC/c complex following K-Ras depletion would be expected to drive cells out of G2. The cell cycle pattern following K-Ras depletion did not show a large decline in the G2 population, and the median for the four replicates without K-Ras depletion was 19.78% (IQR 12.8, 23.74), while, with K-Ras depletion, it was 18.1% (IQR 16.7%, 19.7%). This difference is nonsignificant according to the Mann–Whitney U-Test (*p* = 0.686). The largest effect of K-Ras depletion was a decline in S-phase, which, without K-Ras depletion, was 14.3% (IQR 12.8%, 16.5%); with K-Ras depletion, it was 6.8% (IQR 6.7%, 11.7%), although even this difference did not reach significance on a Mann–Whitney U-Test (*p* = 0.057) (Figure 3a). In the 5-ethynyl-2′-deoxyuridine (EdU) labelling experiments, at the start of labelling, all G2 cells were unlabelled, and only labelled cells will enter G2 from S-phase. At first, unlabelled cells will enter G1 from G2; then, after a period of time, labelled and unlabelled cells will enter G1. There should be a decline in the proportion of cells in G2 that are unlabelled over time, and the proportion of unlabelled cells in G2 should decline (as each unlabelled G2 cell becomes 2 unlabelled G1 cells); however, this decline should slow over time as more of the G2 cells entering G1 become labelled. With the Suit-2 cell line, we noted that the majority of unlabelled G2 cells entered G1 within 18 h of labelling (Figure 3b,c); however, the proportion of G2 cells that are unlabelled remained fairly constant (Figure 3c), suggesting that, while labelled cells can pass easily out of G2 at the same rate that they enter it, unlabelled cells transition much more slowly. As the total proportion of unlabelled cells in G2 is still declining (Figure 3d), there must also be an even slower transition of unlabelled cells through the S-phase than the transition from unlabelled cells from G2 into G1. With the knockdown of K-Ras, the proportion of unlabelled cells in G2 is much higher after 18 h, confirming that the knockdown slows cell cycle progression, including the transition from G2 to G1. The cycle is slowed, but not stopped, and the proportion of G2 cells that are unlabelled gradually declines (Figure 3c). However, the proportion of unlabelled cells that are in G2 (Figure 3d) does not decline, if anything increasing, suggesting that more unlabelled cells are passing through S-phase than are entering G1 from G2. This strongly suggests that K-Ras depletion does not speed up the exit from G2, but rather slows exit from G2 relative to entry into S-phase. This indicates that the decline in G2 cyclins after K-Ras depletion is not associated with an anaphase transition.

### 3.2. K-Ras Depletion Reduces G2 Cyclin Levels by a Proteasome-Mediated Reduction in G2 Cyclin Transcription

**Figure 4:**
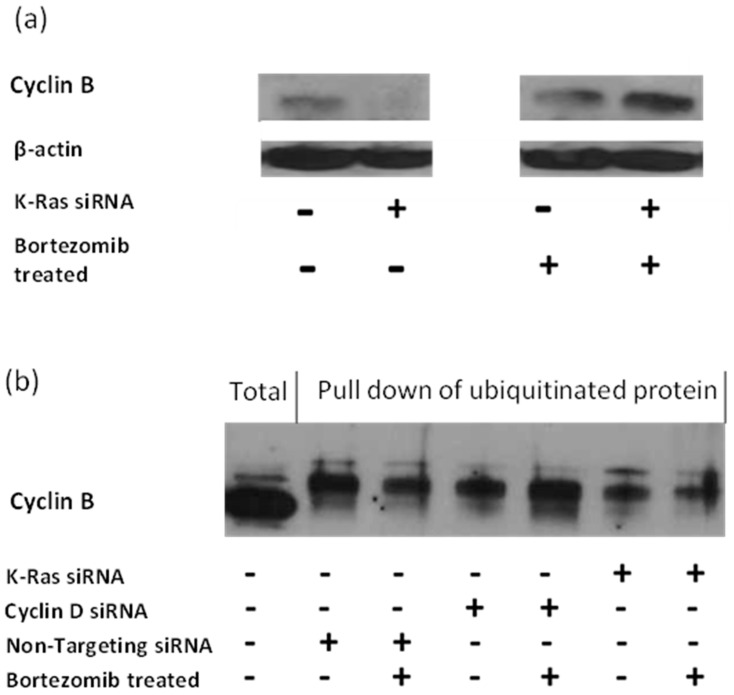
Effect of K-Ras depletion on G2 cyclins with or without Bortezomib treatment: (**a**) Western blot showing the effect of K-Ras depletion on total cyclin B in Suit-2 cells and how bortezomib treatment alters the effect. (**b**) Western blot showing the effect of bortezomib and siRNA in combination.

The APC/c complex targets G2 cyclins to the proteasome via ubiquitination [40]. Therefore, the inhibition of the proteasome with Bortezomib should result in an accumulation of APC/c targets and the increase in protein should be the result of an increase in the ubiquitinated forms of the target. Consistent with this, G2 cyclins levels are increased with Bortezomib treatment, and this is most pronounced following K-Ras depletion, suggesting that the stabilisation of G2 cyclins by K-Ras occurs via a reduction in protein degradation by the proteasome (Figure 4a). However, the level of ubiquitinated G2 cyclin decreased rather than increased after K-Ras depletion (Figure 4b).

K-Ras depletion decreases ubiquitinated cyclin B and bortezomib treatment decreases it further. Cyclin D depletion also decreases ubiquitinated cyclin B; however, bortezomib treatment restores levels.

**Figure 5:**
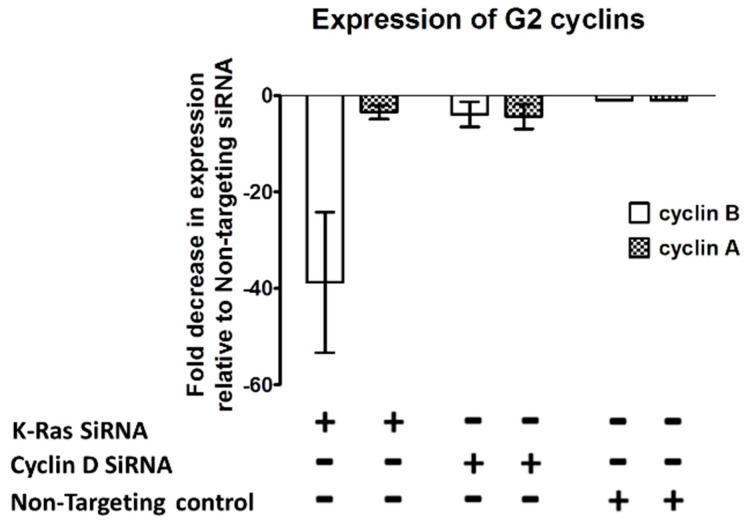
Effect of K-Ras depletion on G2 cyclin transcription. Graph showing an example of the decrease observed in G2 cyclin transcript when K-Ras or cyclin D is depleted. Cyclin B transcript levels after depletion with K-Ras are significantly lower than in non-targeting controls and after depletion with cyclin D (*p* < 0.001, Mann–Whitney U test, standard deviation used as error bar). Cyclin A levels are also significantly lower after depletion of K-Ras than with no depletion; however, there was an equivalent decrease after cyclin D depletion.

If K-Ras depletion does not cause an increase in the proteasomal degradation of G2 cyclins, the question arises as to whether their regulation is at the transcriptional level. Quantitative Reverse Transcriptase Real Time PCR (qRT-PCR) of mRNA from G2 cyclins following K-Ras depletion shows that cyclin B1 was downregulated when K-Ras levels were depleted (Figure 5). This reduction could be explained via reduced G1 to S transition resulting from a failure to upregulate cyclin D1; however, cyclin D1 knockdown did not have as significant an impact on cyclin B1 transcription levels as K-Ras depletion. A reduction in cyclin A2 was less clear than for cyclin B1 and not significantly differ from the reduction in cyclin A2 observed when cyclin D was depleted (Figure 5).

### 3.3. Role of Wild-Type N-Ras in Cell Lines with Mutant K-Ras

**Figure 6:**
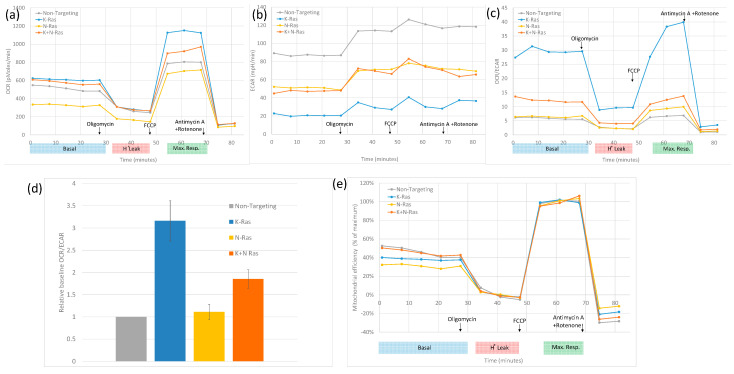
Effects of K-Ras and N-Ras depletion on oxidative phosphorylation: (**a**) Representative Sea Horse analysis showing oxygen consumption rate (OCR) measured over time with the treatments shown. Oligomycin reduced oxygen consumption to just that due to proton leak, while FCCP uncoupled mitochondria resulting in maximal oxygen consumption. K-Ras knockdown caused an increase in uncoupled oxygen consumption while the N-Ras knockdown caused a reduction in maximal and basal oxygen consumption as well as a decrease in proton leak from mitochondria. Knockdown of N-Ras with K-Ras restores basal oxygen consumption with a smaller increase in maximal oxygen consumption and no reduction in proton leak. (**b**) The same experiment but showing Extra Cellular Acidification Rate (ECAR). K-Ras knockdown causes a reduction in ECAR, and N-Ras knockdown alone causes a smaller ECAR reduction, which is equivalent to the ECAR reduction seen when N-Ras and K-Ras are knocked down. (**c**) OCR/ECR is a measure of relative levels of respiration and glycolysis. K-Ras knockdown causes an increase in relative levels of respiration. Repeated experiments have showed this to be significantly greater than the increase seen with both K-Ras and N-Ras knockdown. N-Ras knockdown alone had little overall effect (**d**). (**e**) When OCR/ECAR is adjusted to the maximum increase in active mitochondrial activity (the difference from maximal to proton leak oxygen consumption), N-Ras knockdown was shown to reduce mitochondrial efficiency (Mann–Whitney U test, *p* < 0.001, standard deviation used as error bar); moreover, K-Ras knockdown had less effect on efficiency and the combination of K-Ras with N-Ras knockdown restored mitochondrial efficiency.

N-Ras has been shown to be active in all the human cell lines used in this study (Figure 1). Previous studies have shown that depleting wild-type N-Ras and H-Ras in K-Ras mutant cell lines reduces EGFR responsiveness [33]. Mutant K-Ras is known to reduce oxidative phosphorylation in cancer cells relative to glycolysis [43]; this was confirmed by a metabolic analysis, as shown in Figure 6. Depletion of K-Ras increases the oxygen consumption rate (OCR) relative to the extra cellular acidification rate (ECAR). In Figure 6a, the basal and maximum OCR is higher in the blue K-Ras-depleted trace compared to the grey non-targeting trace, contrasting with a higher ECAR for the non-targeting sample, as shown in Figure 6b. OCR/ECAR is shown in Figure 6c. Although, N-Ras depletion alone had little effect, specifically, a slight increase in OCR/ECAR ratio, when compared to cells treated with control siRNA, OCR and ECAR are lower in the yellow N-Ras-depleted traces than in the non-targeting traces in Figure 6a,b, resulting in a rough equivalence of OCR/ECAR with non-targeting SiRNA treatment, although the N-Ras depletion does result in a slightly higher ratio after FCCP treatment. When both K and N-Ras are depleted, the increase in OCR is significantly less than with the depletion of K-Ras alone (Figure 6d). Further assessment of the data indicates that an increase in OCR/ECAR with K-Ras depletion seems to be due to an increase in maximum mitochondrial activity (Figure 6a) but with no increase in the efficiency of respiration (Figure 6e), the OCR/ECAR increase at baseline being mainly due to a decrease in glycolysis rate (Figure 6b). In contrast, N-Ras depletion causes a reduction in both maximal levels of mitochondrial activity (Figure 6a) and a decrease in the efficiency of respiration (Figure 6e). This suggests that mutant K-Ras reduces both maximal respiration and the efficiency of respiration, compensated in part by an increase in glycolysis, as well as by the mutant K-Ras-dependent activity of N-Ras, which increases the efficiency of respiration and glycolysis.

## 4. Discussion

The role of the activating mutations of K-Ras in tumorigenesis and in the maintenance of cancer is far from clear. Our work confirms that K-Ras depletion reduces the level of APC/c targets (Figure 2) [42], which would fit with mutant K-Ras increasing G2 cyclins by inhibiting APC/c or in general inhibiting proteasomes. This concept was initially supported by the observation that proteasome inhibition negates the decline in G2 cyclins following K-Ras depletion (Figure 4). The inhibition of the proteasome has previously been shown to increase the APC/c targets via an increase in the level of ubiquitinated protein [44]. However, with K-Ras depletion, bortezomib treatment failed to deliver any increase in the ubiquitination of the G2 cyclins, in contrast to the effect of bortezomib following cyclin D1 depletion (where the expected increase in ubiquitinated cyclin B1 was seen). An observed decline in cyclin B1 transcript levels following the loss of K-Ras is evidence that a function of mutant K-Ras is to increase at least this cyclin at a transcriptional level (Figure 5). Transcriptional control of cyclin B1 using K-Ras must be dependent on preventing the degradation of a protein or proteins using the proteasome (Bortezomib increases Cyclin B1 level but not ubiquitinated Cyclin B1 level after K-Ras depletion, Figure 4); however, the affected proteins are as yet undefined.

The effect of mutant K-Ras on increasing cyclins (G2 or otherwise) would be predicted to increase the rate of progression from G1 through S-phase and G2; however, it would, if anything, inhibit anaphase. The depletion of K-Ras would (in this purely cell cycle-centric model) drive cells out of G2 and cause an accumulation of cells in G1.

During progression, cells can become independent of K-Ras in respect to cell survival and cell division. Even Suit-2 (which was previously described as K-Ras-dependent) does not need the continued expression of the oncogene for survival, although its growth rate is severely restricted following K-Ras depletion. K-Ras mutation does drive quiescent cells to divide via G1 cyclins, and EGFR contributes to the increase in G1 cyclins; moreover, in early cancers, it is essential for tumour progression, however further mutations relieve this requirement [23]. Our data also show that, in Suit-2 cells, cyclin D1 levels are unaffected by K-Ras depletion, therefore the transcriptional activation of cyclin D1 by K-Ras is probably of little importance in an established tumour. K-Ras can promote EMT which is important for tumorigenesis; however, again, it may not be important to maintain an established cancer. Indeed, our data supports a K-Ras activity that drives up G2 cyclins, which, if anything, would act to oppose EMT, given the previous reports of cyclin A2’s role in stabilising an epithelial phenotype [41]. Given the reduced growth rate in Suit-2 after K-Ras depletion, it is reasonable to assume that it is K-Ras-driven G2 cyclin production that supports the maintenance of cancer cell division; however, this is not linked to any inhibition of exit from G2 (as would be expected if cyclin levels were maintained at a high level), given that the relative level of exit from G2 compared to exit from G1 is actually decreased through the knockdown of mutant K-Ras (Figure 3). It is convenient to think of two forms of G2 cyclin regulation, with one form being cycle-dependent: G2 cyclins rise in G2 because it is a G2 phase and fall at anaphase (because it is anaphase), and the other form of regulation, which is independent of the cell cycle, in the sense that the G2 cyclins rise due to processes that, in a normal cell, occur in G2 and fall due to processes that occur at anaphase in a normal cell (but which do not in themselves define G2 or anaphase).

At some point in the history of all our human cancer cell lines, N-Ras became active at a basal level. This N-Ras is presumably acting in a wild-type, cell cycle-dependent manner. N-Ras activity is not necessary for an increase in G2 cyclins or indeed for other APC/c targets (Figure 2). N-Ras activity does impact on other functions of K-Ras such as the inhibition of oxidative phosphorylation (Figure 6d). N-Ras depletion alone has little effect on the OCR/ECAR ratio; however, when both K- and N-Ras are lost, the OCR/ECAR ratio is lower than when K-Ras alone is depleted. This can be explained by the K-Ras-dependent activity of N-Ras, which increases both glycolysis and respiration (see Figure 6). This suggests that, during tumorigenesis, the deleterious effects of K-Ras are offset by the selection of cells, which can produce wild-type N-Ras, and that K-Ras mutation causes N-Ras activity that compensates for the mutation. In a mouse tumour where mutant K-Ras is endogenously expressed alongside mutant p53, N-Ras basal activity does not appear to be necessary for tumour maintenance (Figure 1b). This may be due to the artificial nature of the genesis of the mouse-derived tumour, which may not have progressed through all the aberrations and alterations that are normally associated with the stepwise tumour development seen naturally.

**Figure 7:**
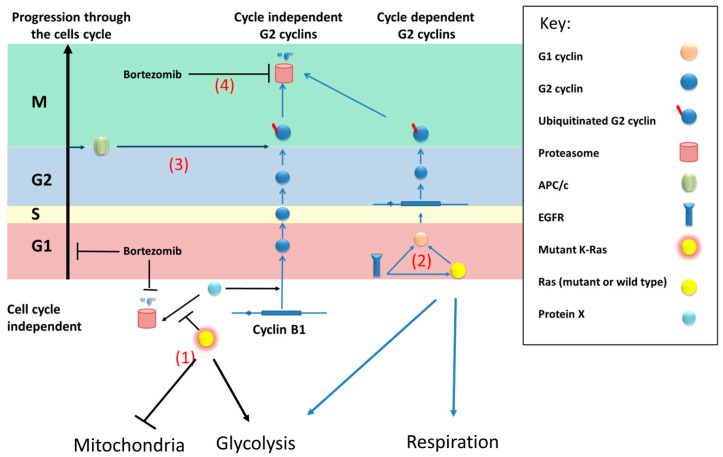
Model of a compensatory mechanism of wild-type Ras expression in cells with mutant K-Ras: (1) Mutant K-Ras causes reduced mitochondrial activity (possibly by reducing the number of mitochondria) and promotes the production of G2 cyclins by inhibition of the proteolysis of an unknown transcriptional activator (x) of cyclin B1. (2) Wild-type (or mutant) Ras will increase transcription of G2 cyclins via G1 cyclins in a cell cycle-dependent fashion. EGFR can activate this process but can also increase G1 cyclins by directly binding to their promoters, compensating for the non-cyclic levels linked to the mutant protein. Furthermore, based on results from N-Ras knockdown, the wild type (N-Ras) increases glycolysis and the efficiency of respiration. (3) G2 cyclins will be degraded via the APC/c complex, therefore, in the absence of mutant Ras, the level of the G2 cyclins will be limited by the proportion of cells in G2. (4) Bortezomib inhibits the proteasome, causing stabilisation of ubiquitinated G2 cyclins, reducing the production of ubiquitinated protein by restricting entry to (and exit from) G2.

We therefore propose a model (Figure 7) where an unknown protein (referred to as protein X) activates the transcription of cyclin B1 (and possibly other G2 cyclins) in a cell cycle-independent fashion. This protein(s) is normally degraded by the proteasome; however, mutant K-Ras directly or indirectly prevents this degradation, for example, by inhibiting its ubiquitination.

Given the importance of the tumour microenvironment in PDAC tumorigenesis and progression, it should be emphasised as a limitation that this current study is based solely on the in vitro data of monocultured PDAC tumour cells.

## 5. Conclusions

In summary, we have observed that mutant K-Ras promotes an increase in G2 cyclins that is not accompanied by any inhibition of exit from G2. This appears to be at least in part via a form of transcriptional regulation which is proteasome-related. We have shown that the activation of wild-type N-Ras is a common event in pancreatic cancer cell lines, which impacts on their metabolism. N-Ras acts in an antagonistic fashion in the regulation of oxidative phosphorylation and that N-Ras does not influence the transcriptional regulation of G2 cyclins.

## Data Availability

Raw data available on request, processed data included in manuscript.
